# Supplementary material for: Exploring the impact of vibrational cavity coupling strength on ultrafast CN + c-C6H12 reaction dynamics
Source: Nanophotonics. 2024 Jan 25;13(14):2591–9. doi: 10.1515/nanoph-2023-0747 (PMC11635944; doi:10.1515/nanoph-2023-0747)
Supplement: Supplementary file 1 — Supplementary Material Details [file j_nanoph-2023-0747_suppl_001.pdf]

- SUPPLEMENTARY MATERIAL -

Exploring the impact of vibrational cavity coupling strength on ultrafast CN + *c*-C_6H_{12} reaction dynamics

Liying Chen,[1] Ashley P. Fidler,[1] Alexander M. McKillop,[1] and Marissa L. Weichman[1,*]

[1]Department of Chemistry, Princeton University, Princeton, New Jersey 08544, USA

[*]weichman@princeton.edu

Section S1: Wavelength-dependent mirror reflectivity and transmission

We use custom-coated distributed Bragg reflector (DBR) mirrors (UltraFast Innovations GmbH) engineered with alternating layers of high and low refractive index materials (HfO_2, SiO_2). The design allows for optical transmission of ultraviolet and visible light (Fig. S1AB) while maintaining high reflectivity in the C−H stretching region (Fig. S1CD). We assess the DBR mirror reflectivity profile using an infrared imaging microscope (Nicolet iN10 Mx, Thermo Scientific). These mirrors display a narrow band of high reflectivity ($R > 90\%$) in the C−H stretching region spanning ~ 2800 – 3400 cm^{-1} (Fig. S1C). Upon assembly into an air-filled 56 μm Fabry–Pérot cavity, we observe a cavity transmission spectrum featuring ~6 cm^{-1} FWHM cavity fringes separated by a ~100 cm^{-1} FSR in the C–H stretching region (Fig. S1D).

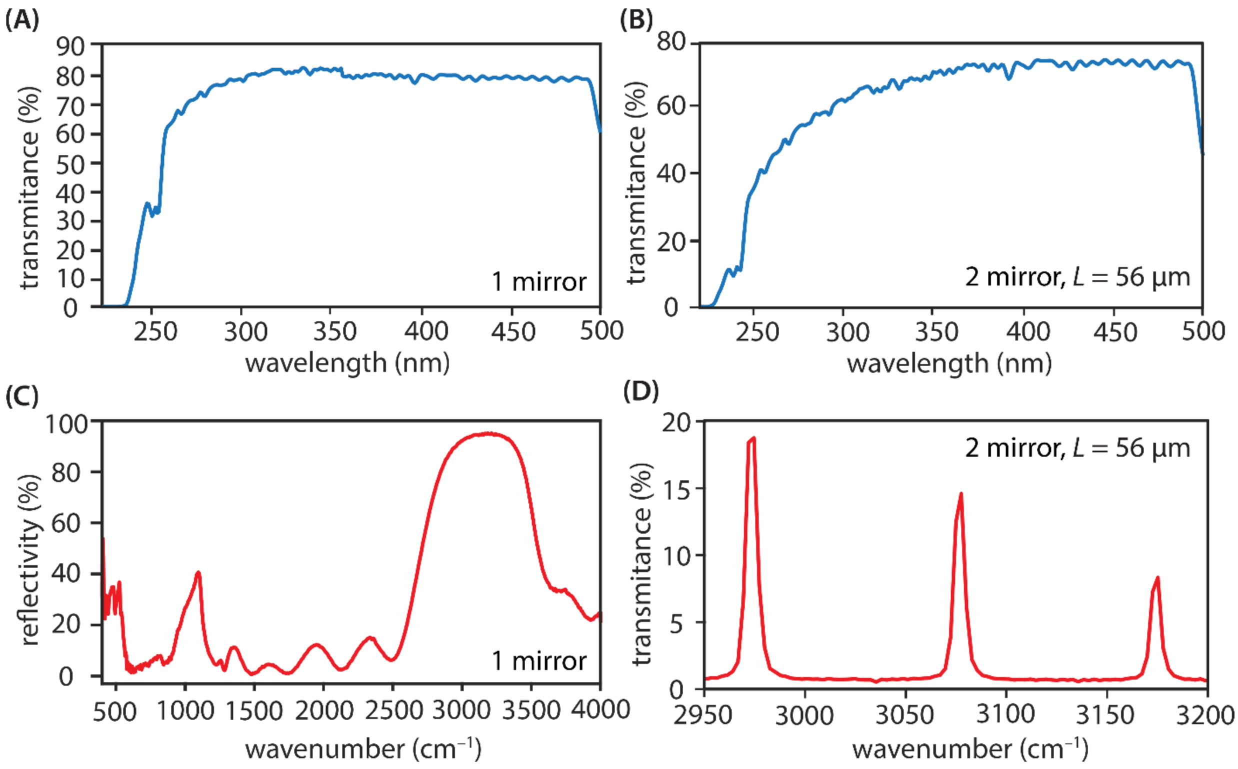

Fig. S1: **(A)** UV-vis transmission spectrum for a single DBR cavity mirror. **(B)** UV-vis transmission spectrum for an air-filled cavity constructed from two mirrors separated by a 56 μm spacer. **(C)** IR reflectivity spectrum of a single DBR mirror. **(D)** IR transmission spectrum of the C–H stretching region obtained for a 56 μm two-mirror cavity.

Table S1: Experimental Rabi splittings obtained for the *c*-C_6H_{12} C–H stretching mode at 2927 cm^{-1} in each *c*-C_6H_{12}:$CHCl_3$ solvent mixture studied here. Rabi splittings are extracted from the corresponding cavity transmission spectra in Fig. 2A of the main text.

***c*-C_6H_{12}:$CHCl_3$ volume ratio**	***c*-C_6H_{12} molarity (M)**	**Rabi splitting (cm^{-1})**
2:20	0.84	55
4:20	1.54	66
6:20	2.13	76
7:20	2.40	80
8:20	2.64	85

Section S2: Experimental and simulated cavity dispersion plots for *c*-C_6H_{12}:$CHCl_3$ mixtures

In Fig. S2, we compare the experimental and simulated transmission spectra for a cavity containing a 2.64 M solution of *c*-C_6H_{12} in $CHCl_3$ plotted as a function of cavity length to show dispersion. We tune the cavity length by translating the assembly in the plane of the cavity and relying on the imperfect parallelism of the cavity mirrors. Experimental cavity lengths are determined by finding the best match between the experimental and simulated dispersion curves. Disparities between the experimental and simulated curves are chiefly due to broadening of the experimental cavity transmission features. We attribute this broadening to the finite resolution of our spectrometer and variations in cavity length within the experimental IR focal volume.

Both experimental and simulated dispersion curves display avoided crossing features, indicative of vibrational strong coupling (VSC) of various C−H stretching modes of both *c*-C_6H_{12} and $CHCl_3$. For the on-resonance cavity-coupling conditions discussed throughout this manuscript, we target VSC of the brightest C-H stretching mode of *c*-C_6H_{12} centered at 2927 cm^{-1} in order to attain the largest Rabi splitting. This on-resonance coupling condition is indicated by the red vertical lines in Fig. S2. Given the spectral congestion inherent in the C−H stretching region of this system, it is challenging to identify a clear off-resonance condition where no cavity mode is coupled to any C−H stretching feature. Here, we use the condition marked by the orange vertical lines in Fig S2 as our off-resonance configuration. We emphasize that this condition may still involve some detuned coupling to the neighboring C−H stretching mode of $CHCl_3$. However, coupling of $CHCl_3$ is not expected to impact any reaction rates, as found in our previous report [1].

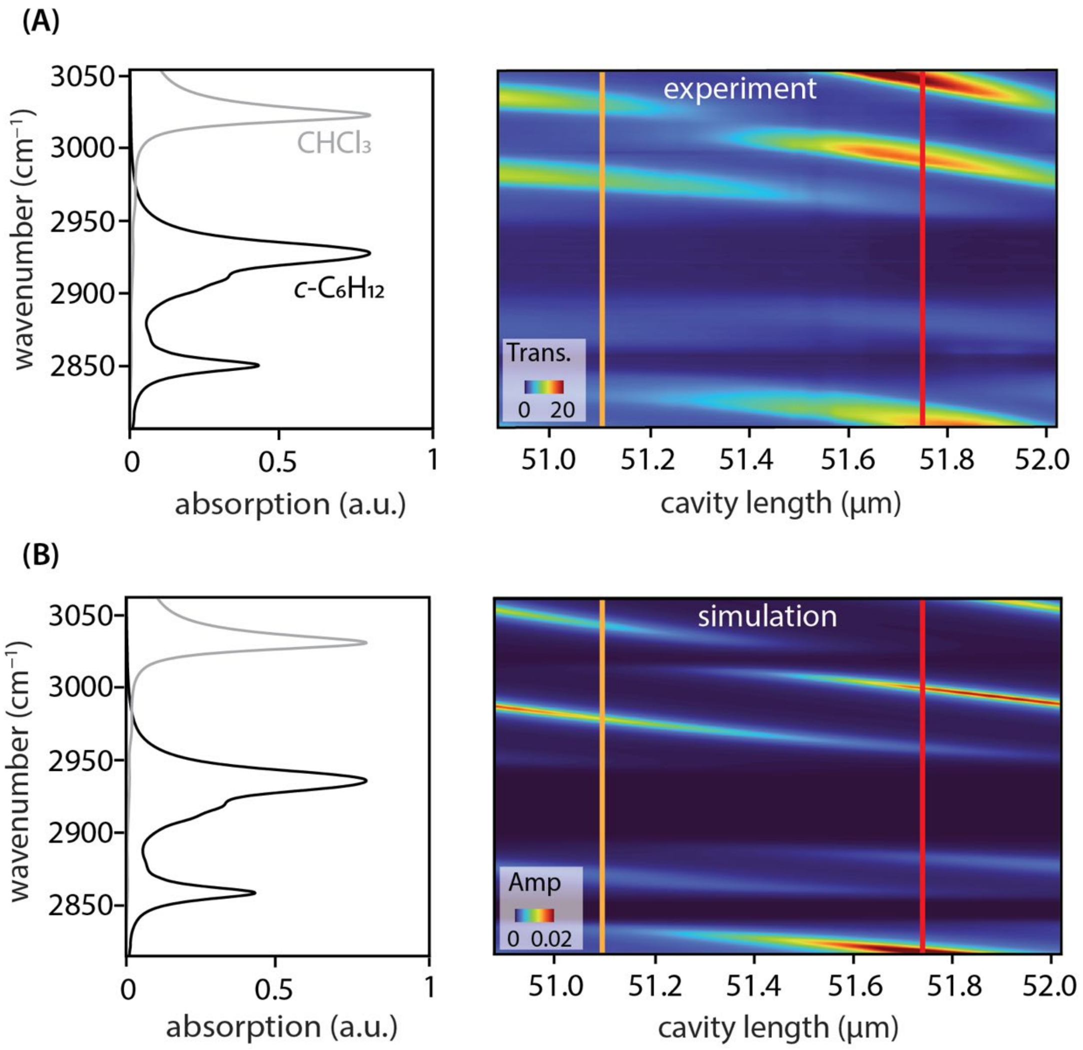

Fig. S2: **(A)** Experimental and **(B)** simulated DBR Fabry-Pérot cavity transmission spectra showing dispersion as a function of cavity length for the 2.64 M *c*-C_6H_{12} solution in $CHCl_3$. The absorption spectra of both *c*-C_6H_{12} (black) and $CHCl_3$ (grey) are plotted in the left-hand panels using reference data from PNNL [2]. The vertical lines drawn on the dispersion plots in the right-hand panels illustrate the on-resonance (red) and off-resonance (orange) conditions.

Section S3: Classical simulation of cavity transmission spectra

To simulate experimental cavity transmission spectra, we use the classical expression for light transmitted through a two-mirror Fabry-Pérot optical cavity [3-5]:

$$\frac{I_T(\nu)}{I_0} = \frac{T(\nu)^2 e^{-\alpha(\nu)L}}{1+R(\nu)^2 e^{-2\alpha(\nu)L} - 2R(\nu)e^{-\alpha(\nu)L}\cos\left(\frac{4\pi L n(\nu)\nu}{c}\right)} \tag{1}$$

Here, $T(\nu)$ and $R(\nu)$ are the frequency-dependent transmittance and reflectance for a single cavity mirror, $\alpha(\nu)$ and $n(\nu)$ are the absorption coefficient and real component of the refractive index for the intracavity material, and L is the cavity length. Reference data for the real ($n(\nu)$) and imaginary ($k(\nu)$) components of the complex refractive indices of *c*-C_6H_{12} and $CHCl_3$ were provided by Pacific Northwest National Laboratory (PNNL) [2]. We convert $k(\nu)$ to absorption coefficient $\alpha(\nu)$ using:

$$\alpha(\nu) = 4\pi k(\nu)\nu \tag{2}$$

Section S4: Reproducibility of intracavity and extracavity transient absorption measurements

Ensuring the robustness of intracavity experimental results is crucial in the context of the well-documented reproducibility challenges in the field of polariton chemistry. We collected multiple independent transient absorption (TA) datasets for each cavity-coupling condition and for each *c*-C_6H_{12}:$CHCl_3$ solvent mixture. We extract time constants from each TA dataset following the fitting methods outlined in the main text. We present all time constants for individual TA scans in 0.84 M, 1.54 M, 2.13 M, 2.40 M, and 2.64 M solutions of *c*-C_6H_{12} in Tables S2-S6.

In processing the datasets summarized in Tables S2-S6, we discard time constants based on two criteria. First, a time constant is discarded if the regression p-value exceeds 0.05, indicating poor spectral fitting during the regression. Secondly, we discard any time constants that fall more than three standard deviations from the mean for a specific cavity condition and *c*-C_6H_{12} concentration. In some datasets, specific time constants could not be clearly resolved and were consequently excluded using the mentioned criteria. Given that the multivariate regression method treats each input component independently, we retained the other time constants in these datasets.

Table S2: Time constants obtained for the CN + *c*-C_6H_{12} reaction in an 0.84 M solution of *c*-C_6H_{12} in $CHCl_3$ under extracavity, on-resonance, and off-resonance conditions.

**0.84 M *c*-C_6H_{12}**	**390 nm** τ_1 (ps)	**340 nm** τ_1 (ps)	τ_2 (ps)
extracavity			
2023-06-14 #1	7.9	2.2	360
2023-06-14 #2	8.7	2.1	360
2023-06-14 #3	7.2	2.4	410
2023-06-14 #4	6.5	2.1	320
2023-06-14 #5	7.4	1.8	300
**Mean**	**7.5 ± 0.8**	**2.1 ± 0.2**	**350 ± 40**
on-resonance			
2023-06-09 #1	7.3	2.0	410
2023-06-09 #2	8.1	1.6	320
2023-06-09 #3	9.6	1.3	380
2023-06-09 #4	8.4	1.7	390
2023-06-09 #5	6.2	1.9	380
**Mean**	**7.9 ± 1.2**	**1.7 ± 0.3**	**380 ± 30**
off-resonance			
2023-06-09 #1	6.5	1.5	460
2023-06-09 #2	8.1	2.0	430
2023-06-09 #3	6.9	1.5	480
2023-06-09 #4	8.0	1.7	410
2023-06-09 #5	5.6	2.0	360
**Mean**	**7.0 ± 1.1**	**1.7 ± 0.3**	**430 ± 50**

Table S3: Time constants obtained for the CN + c-C_6H_{12} reaction in a 1.54 M solution of c-C_6H_{12} in $CHCl_3$ under extracavity, on-resonance, and off-resonance conditions. Missing time constants were discarded due to poor fitting, as described in Section S3.

	**390 nm**	**340 nm**	
**1.54 M *c*-C_6H_{12}**	τ_1 (ps)	τ_1 (ps)	τ_2 (ps)
extracavity			
2023-06-16 #1	5.5	1.78	390
2023-06-16 #2	4.8	1.50	340
2023-06-16 #3	5.0	1.62	280
2023-06-16 #4	5.3	1.67	340
2023-06-16 #5	5.0	1.76	410
**Mean**	**5.1 ± 0.3**	**1.67 ± 0.12**	**350 ± 50**
on-resonance			
2023-06-13 #1	4.4	1.15	400
2023-06-13 #2	5.3		
2023-06-13 #3	4.5	1.11	450
2023-06-13 #4	5.3	1.33	460
2023-06-13 #5	5.6	1.31	400
**Mean**	**5.0 ± 0.5**	**1.23 ± 0.11**	**430 ± 30**
off-resonance			
2023-06-13 #1	5.2	1.13	400
2023-06-13 #2	4.8	1.30	390
2023-06-13 #3	4.4	1.41	400
2023-06-13 #4	4.9	1.30	510
2023-06-13 #5	5.1	1.05	370
**Mean**	**4.9 ± 0.3**	**1.24 ± 0.15**	**410 ± 60**

Table S4: Time constants obtained for the CN + *c*-C_6H_{12} reaction in a 2.13 M solution of *c*-C_6H_{12} in $CHCl_3$ under extracavity, on-resonance, and off-resonance conditions. Missing time constants were discarded due to poor fitting, as described in Section S3.

	**390 nm**	**340 nm**	
**2.13 M *c*-C_6H_{12}**	τ_1 (ps)	τ_1 (ps)	τ_2 (ps)
extracavity			
2023-06-20 #1	5.7	1.4	330
2023-06-20 #3	4.6	1.5	260
2023-06-20 #4	4.6	2.3	280
2023-06-20 #5	4.9	1.7	280
**Mean**	**4.9 ± 0.5**	**1.7 ± 0.4**	**280 ± 30**
on-resonance			
2023-06-21 #1	4.9	1.1	470
2023-06-21 #2	4.9	1.1	
2023-06-21 #3	4.9		
2023-06-21 #4	3.7	1.0	250
2023-06-21 #5	4.1	1.7	310
**Mean**	**4.5 ± 0.6**	**1.2 ± 0.3**	**340 ± 110**
off-resonance			
2023-06-21 #1	4.76	1.3	260
2023-06-21 #2	4.82	1.9	
2023-06-21 #3	4.50	1.3	310
2023-06-21 #4	4.72	1.8	320
2023-06-21 #5	4.85		330
**Mean**	**4.73 ± 0.14**	**1.6 ± 0.3**	**300 ± 30**

Table S5: Time constants obtained for the CN + c-C_6H_{12} reaction in a 2.40 M solution of c-C_6H_{12} in $CHCl_3$ under extracavity, on-resonance, and off-resonance conditions.

**2.40 M c-C_6H_{12}**	**390 nm** τ_1 (ps)	**340 nm** τ_1 (ps)	**340 nm** τ_2 (ps)
extracavity			
2023-07-05 #2	3.7	1.4	250
2023-07-05 #3	3.2	1.6	230
2023-07-05 #5	4.1	1.5	290
2023-07-05 #6	4.3	1.3	340
2023-07-05 #7	3.8	1.2	370
2023-07-05 #8	3.6	1.4	390
2023-07-05 #9	3.7	1.5	350
2023-07-05 #10	4.1	1.6	330
2023-07-05 #11	4.1	1.5	340
**Mean**	**3.8 ± 0.3**	**1.7 ± 0.9**	**320 ± 60**
on-resonance			
2023-07-04 #1	2.7	1.0	200
2023-07-04 #2	4.3	1.5	560
2023-07-04 #3	5.8	2.0	240
2023-07-04 #4	4.9	1.1	280
2023-07-04 #5	4.0	1.9	380
2023-07-04 #6	4.6	1.0	440
**Mean**	**4.4 ± 1.0**	**1.4 ± 0.4**	**350 ± 140**
off-resonance			
2023-07-04 #1	4.6	1.0	230
2023-07-04 #2	4.9	1.0	430
2023-07-04 #3	3.6	1.0	280
2023-07-04 #4	4.3	1.5	170
2023-07-04 #5	4.0	1.4	360
2023-07-04 #6	4.2	1.3	320
**Mean**	**4.2 ± 0.5**	**1.2 ± 0.2**	**300 ± 90**

Table S6: Time constants obtained for the CN + c-C_6H_{12} reaction in a 2.64 M solution of c-C_6H_{12} in $CHCl_3$ under extracavity, on-resonance, and off-resonance conditions.

	**390 nm**	**340 nm**	
**2.64 M c-C_6H_{12}**	τ_1 (ps)	τ_1 (ps)	τ_2 (ps)
extracavity			
2023-07-11 #1	2.8	1.3	220
2023-07-11 #2	3.3	1.9	230
2023-07-11 #3	3.7	1.7	240
2023-07-12 #1	3.0	1.4	290
2023-07-12 #2	3.9	1.5	330
2023-07-12 #3	3.1	1.4	280
2023-07-12 #5	2.8	1.7	190
**Mean**	**3.2 ± 0.4**	**1.6 ± 0.2**	**250 ± 50**
on-resonance			
2023-07-13 #1	3.5	1.3	350
2023-07-14 #2	3.0	1.8	290
2023-07-14 #3	3.0	1.0	130
2023-07-18 #1	3.9	1.1	430
2023-07-18 #4	3.8	1.6	390
2023-07-18 #5	3.0	1.6	430
2023-07-19 #3	3.6	1.6	260
2023-07-19 #6	3.5	1.5	280
2023-07-20 #1	3.3	1.4	250
2023-07-20 #2	3.4	2.0	270
2023-07-20 #3	2.9	1.6	370
2023-07-20 #4	3.1	1.4	220
2023-07-20 #5	3.3	1.5	290
2023-07-20 #6	2.7	1.1	290
2023-07-21 #1	2.9	1.5	490
2023-07-21 #2	3.7	1.9	280
2023-07-21 #3	3.8	2.0	440
**Mean**	**3.4 ± 0.4**	**1.5 ± 0.3**	**320 ± 90**

... Table S6 continued on next page

... *Table S6 continued from previous page*

**2.64 M C_6H_{12}**	**390 nm** τ_1 (ps)	**340 nm** τ_1 (ps)	τ_2 (ps)
off-resonance			
2023-07-14 #1	3.0	1.6	230
2023-07-14 #3	3.3	1.5	230
2023-07-18 #1	2.5	1.0	360
2023-07-18 #4	3.1	1.2	420
2023-07-18 #5	3.6	1.8	230
2023-07-19 #2	3.1	1.4	410
2023-07-19 #3	3.1	1.4	210
2023-07-19 #8	2.6	1.5	210
2023-07-20 #1	3.4	1.6	240
2023-07-20 #2	2.7	1.4	270
2023-07-20 #3	3.4	1.6	240
2023-07-20 #4	3.3	1.7	280
2023-07-20 #5	3.5	1.7	260
2023-07-20 #6	3.5	1.3	230
2023-07-21 #1	3.6	1.8	170
2023-07-21 #2	3.3	1.7	170
2023-07-21 #3	3.6	1.9	240
**Mean**	**3.2 ± 0.4**	**1.5 ± 0.2**	**260 ± 70**

References

[1] A. P. Fidler, L. Chen, A. M. McKillop, and M. L. Weichman, "Ultrafast dynamics of CN radical reactions with chloroform solvent under vibrational strong coupling," *J. Chem. Phys.,* vol. 159, no. 16, p. 164302, 2023, doi: 10.1063/5.0167410.

[2] T. Myers *et al.*, "Accurate optical constants for liquids in the near-infrared: Improved methods for obtaining the *n* and *k* constants from 1 to 4 μm," *Chemical, Biological, Radiological, Nuclear, and Explosives (CBRNE) Sensing XX,* vol. 110100, pp. 138-144, 2019, doi: 10.1117/12.2519499.

[3] Y. F. Zhu, D. J. Gauthier, S. E. Morin, Q. L. Wu, H. J. Carmichael, and T. W. Mossberg, "Vacuum Rabi splitting as a feature of linear-dispersion theory: Analysis and experimental observations," *Phys. Rev. Lett.,* vol. 64, no. 21, pp. 2499-2502, 1990, doi: 10.1103/PhysRevLett.64.2499.

[4] W. Nagourney, *Quantum Electronics for Atomic Physics and Telecommunication*, 2nd ed., Oxford, UK: Oxford University Press, 2014.

[5] B. S. Simpkins, A. D. Dunkelberger, and I. Vurgaftman, "Control, modulation, and analytical descriptions of vibrational strong coupling," *Chem. Rev.,* vol. 123, no. 8, pp. 5020-5048, 2023, doi: 10.1021/acs.chemrev.2c00774.
